# Supplementary material for: Is tamoxifen good enough for the Asian population in ER+ HER2- post-menopausal women with early breast cancer? A nationwide population-based cohort study
Source: PLoS One. 2024 Nov 27;19(11):e0313120. doi: 10.1371/journal.pone.0313120 (PMC11602078; doi:10.1371/journal.pone.0313120)
Supplement: Attachment — Submitted filename: Response to Reviewers.docx [file pone.0313120.s001.docx]

Response to Reviewers

From: Journal Requirements

|  | Journal Requirements | Response to Reviewers |
| --- | --- | --- |
| 1 | Please ensure that your manuscript meets PLOS ONE's style requirements, including those for file naming. | I will ensure the manuscript complies with PLOS ONE's style requirements, including file naming. |
| 2 | consider depositing your raw data in a repository to ensure your work is read, appreciated and cited by the largest possible audience. | **Availability of data and materials:** The data supporting this study's findings are available from the Health and Welfare Data Center (HWDC) established by Taiwan's Ministry of Health and Welfare. However, restrictions apply to the availability of these data, which were used under license for the present study and are, thus, not publicly available. The data may be accessed by applying to the Health and Welfare Data Center (HWDC, https://www.apre.mohw.gov.tw/). |
| 3 | Please state what role the funders took in the study. | I will include the following information in the cover letter. "The funders had no role in study design, data collection and analysis, decision to publish, or preparation of the manuscript." |
| 4 | Please note that funding information should not appear in the Acknowledgments section or other areas of your manuscript. | Thank you for your correction. The funding information has been removed from the Acknowledgments section and will not appear anywhere in the manuscript. |
| 5 | We note that you have indicated that there are restrictions to data sharing for this study. PLOS only allows data to be available upon request if there are legal or ethical restrictions on sharing data publicly. For more information on unacceptable data access restrictions, please see <http://journals.plos.org/plosone/s/data-availability#loc-unacceptable-data-access-restrictions>.  Before we proceed with your manuscript, please address the following prompts:  a) If there are ethical or legal restrictions on sharing a de-identified data set, please explain them in detail (e.g., data contain potentially identifying or sensitive patient information, data are owned by a third-party organization, etc.) and who has imposed them (e.g., a Research Ethics Committee or Institutional Review Board, etc.). Please also provide contact information for a data access committee, ethics committee, or other institutional body to which data requests may be sent.  b) If there are no restrictions, please upload the minimal anonymized data set necessary to replicate your study findings to a stable, public repository and provide us with the relevant URLs, DOIs, or accession numbers. For a list of recommended repositories, please see  <https://journals.plos.org/plosone/s/recommended-repositories>. You also have the option of uploading the data as Supporting Information files, but we would recommend depositing data directly to a data repository if possible.  We will update your Data Availability statement on your behalf to reflect the information you provide. | The Ministry of Health and Welfare in Taiwan, while ensuring the protection of individual health privacy, promotes the sharing of health information to derive aggregated information of practical value. This facilitates the enhancement of public health decision-making quality and the advancement of academic research. Health and welfare data utilization in this study is categorized as the most stringent tier, requiring compliance with specific management guidelines. Processing can only take place in designated physical workspaces within the Ministry of Health and Welfare, where databases can be linked, and only statistical outcomes can be extracted for use. The data may be accessed by applying to the Health and Welfare Data Center (HWDC, <https://www.apre.mohw.gov.tw/>); the project director submitting this application must be a Taiwanese national. Data are, however, available from the authors upon reasonable request to contact Miss. Wu (stamy918@mohw.gov.tw). |
| 6 | Please amend your manuscript to include your abstract after the title page. | An abstract has been added following the title page. |
| 7 | Please upload a new copy of Figure 2 as the detail is not clear. | To address the issue of unclear details, we will separate Figure 2(A) and 2(B) into two separate files for uploading. |

From: Reviewers' comments

|  | Reviewer's Responses to Questions | Response to Reviewers |
| --- | --- | --- |
| 1 | Is the manuscript technically sound, and do the data support the conclusions? The manuscript must describe a technically sound piece of scientific research with data that supports the conclusions. Experiments must have been conducted rigorously, with appropriate controls, replication, and sample sizes. The conclusions must be drawn appropriately based on the data presented.  Reviewer #1: Partly | Thank you very much for your valuable feedback on this study. We will promptly make the necessary revisions to improve our research. |
| 2 | Has the statistical analysis been performed appropriately and rigorously?  Reviewer #1: Yes | Thank you. |
| 3 | Have the authors made all data underlying the findings in their manuscript fully available? The [PLOS Data policy](http://www.plosone.org/static/policies.action#sharing) requires authors to make all data underlying the findings described in their manuscript fully available without restriction, with rare exception (please refer to the Data Availability Statement in the manuscript PDF file). The data should be provided as part of the manuscript or its supporting information, or deposited to a public repository. For example, in addition to summary statistics, the data points behind means, medians and variance measures should be available. If there are restrictions on publicly sharing data—e.g. participant privacy or use of data from a third party—those must be specified.  Reviewer #1: No | 1. The data supporting this study's findings are available from the Health and Welfare Data Center (HWDC, <https://www.apre.mohw.gov.tw/>) established by Taiwan's Ministry of Health and Welfare. However, restrictions apply to the availability of these data, which were used under license for the present study and are, thus, not publicly available. Health and welfare data utilization in this study is categorized as the most stringent tier, requiring compliance with specific management guidelines. Processing can only take place in designated physical workspaces within the Ministry of Health and Welfare, where databases can be linked, and only statistical outcomes can be extracted for use. The data may be accessed by submitting an application to the HWDC; the project director submitting this application must be a Taiwanese national. Data are, however, available from the authors upon reasonable request to contact Miss. Wu ([stamy918@mohw.gov.tw](mailto:stamy918@mohw.gov.tw)). 2. Not only aggregate numbers but also means, variance, medians, minimum, and maximum values are available. |
| 4 | Is the manuscript presented in an intelligible fashion and written in standard English? Reviewer #1: Yes | Thank you. |
| 5 | - The section on age for postmenopausal status can be shortened. | Following the Reviewer's suggestions, I have condensed this passage. After paraphrasing, the content has been reduced from 169 to 109 words. (Page 7, line 18 to Page 8, line 8.) |
|  | - The follow-up is short to evaluate the true effect of any endocrine therapy, this should be added to the discussion section. The difference in outcome by type of endocrine therapy is hard to evaluate after less than five years of follow-up. - Please also discuss a rather short follow-up time as a weakness of the study. | We appreciate the reviewer's valuable suggestions for our study. The incidences, with each individual being observed for at least 4 years, were recorded in the registry from 2012 to 2016 and assessed until the end of 2019. Recognizing this limitation, the present study revises the definition of the outcome in the manuscript from deaths to recurrence. In the discussion section, we will address the shorter follow-up period as a limitation of our research. (Page 25, line 14 to Page 26, line 3.) |
|  | - Data on adjuvant chemotherapy should be included as well as data on the tumor´s biological inherence. A minimum is data on oestrogen receptor, HER2-receptor and some proliferation estimation such as Ki67 or histological grade. All these factors greatly influence outcome and it was early anticipated that tumor biology could distinguish breast cancer populations who had greater benefit of adjuvant aromatase inhibitors. This variables should also be discussed in the Discussion. A sentence on Taiwan´s nationel guidelines for adjuvant therapy would also be helpful for the reader. - as an obtainable patient and tumor information, data on adjuvant chemotherapy and predictive tumor characteristics (ER and HER2 status, proliferation markers, histological grade) should be presented and discussed in the light of tumor responses. | We appreciate the suggestions provided by the reviewers, which have enhanced the comprehensiveness of our study. As recommended, we have included five additional variables in our analysis—adjuvant chemotherapy, estrogen receptor, HER2 receptor, Ki67, and histological grade. Among these variables, data for adjuvant chemotherapy and histological grade were available for direct analysis as they were purchased. We did not purchase data for estrogen receptors and HER2 receptors. However, given our inclusion criteria requiring patients to have received hormonal therapy for at least one year, we can assume positive estrogen receptor status.  Additionally, we used whether patients received targeted therapy as a proxy for HER2 receptor status. The enrollment period for our study was from 2012 to 2016, and data for Ki67 were available from 2018 from our hospital's Cancer Registry. Therefore, it is inferred that there would be a significant proportion of missing data for Ki67 in the Taiwan Cancer Registry from 2012 to 2016, limiting the study's value. In conclusion, we have incorporated adjuvant chemotherapy, radiation therapy, targeted therapy, and histological grade as variables in our study. |
|  | - Consider using invasive recurrence free survival or interval instead of overall survival; these endpoints are more relevant as an endpoint in breast cancer trials / retrospective studies. - use of invasive-free survival instead of overall survival would be advisable as suggested. | Following the reviewer's suggestion, we have changed the endpoint from overall survival to recurrence-free survival. The occurrence of recurrence in the Tamoxifen group of stage 3 breast cancer patients was limited to one person, which precludes further statistical analysis. Therefore, the inclusion criteria have been revised from breast cancer stages 1-3 to stages 1-2. The number of participants has been adjusted from 9,180 individuals to 7,812 individuals. Due to the change in the endpoint, the "Materials and Methods" and "Results" sections in the revised manuscript will also be amended accordingly. |
|  | - The number of patients at risk should be listed in the Table of outcome by stage. | Add new Table 2 (Page 16, line 13.) to show the number of patients with recurrence at different stages of breast cancer. |
| 6 | PLOS authors have the option to publish the peer review history of their article ([what does this mean?](https://journals.plos.org/plosone/s/editorial-and-peer-review-process#loc-peer-review-history)). If published, this will include your full peer review and any attached files.  If you choose “no”, your identity will remain anonymous but your review may still be made public.  **Do you want your identity to be public for this peer review?** For information about this choice, including consent withdrawal, please see our [Privacy Policy](https://www.plos.org/privacy-policy).  Reviewer #1: **Yes:** Lisa Rydén, professor Lund University | Yes. |
