# Supplementary material for: Is tamoxifen good enough for the Asian population in ER+ HER2- post-menopausal women with early breast cancer? A nationwide population-based cohort study
Source: PLoS One. 2024 Nov 27;19(11):e0313120. doi: 10.1371/journal.pone.0313120 (PMC11602078; doi:10.1371/journal.pone.0313120)
Supplement: Attachment — Submitted filename: Response to Reviewers.docx [file pone.0313120.s002.docx]

**Response to Reviewers**

|  | **Reviewers' comments** | **Response to Reviewers** |
| --- | --- | --- |
| 1 | If the authors have adequately addressed your comments raised in a previous round of review and you feel that this manuscript is now acceptable for publication, you may indicate that here to bypass the“Comments to the Author”section, enter your conflict of interest statement in the“Confidential to Editor”section, and submit your "Accept" recommendation.  Reviewer #1: (No Response) | - |
| 2 | Is the manuscript technically sound, and do the data support the conclusions?  The manuscript must describe a technically sound piece of scientific research with data that supports the conclusions. Experiments must have been conducted rigorously, with appropriate controls, replication, and sample sizes. The conclusions must be drawn appropriately based on the data presented.  Reviewer #1: Partly | Thank you very much for your valuable feedback on this study. We will promptly make the necessary revisions to improve our research. |
| 3 | Has the statistical analysis been performed appropriately and rigorously?  Reviewer #1: Yes | Thank you. |
| 4 | Have the authors made all data underlying the findings in their manuscript fully available?  The [PLOS Data policy](http://www.plosone.org/static/policies.action#sharing) requires authors to make all data underlying the findings described in their manuscript fully available without restriction, with rare exception (please refer to the Data Availability Statement in the manuscript PDF file). The data should be provided as part of the manuscript or its supporting information, or deposited to a public repository. For example, in addition to summary statistics, the data points behind means, medians and variance measures should be available. If there are restrictions on publicly sharing data—e.g. participant privacy or use of data from a third party—those must be specified.  Reviewer #1: Yes | Thank you. |
| 5 | Is the manuscript presented in an intelligible fashion and written in standard English?  PLOS ONE does not copyedit accepted manuscripts, so the language in submitted articles must be clear, correct, and unambiguous. Any typographical or grammatical errors should be corrected at revision, so please note any specific errors here.  Reviewer #1: Yes | Thank you. |
| 6 | Review Comments to the Author  Reviewer #1: Thank you for the updated manuscript. |  |
| 6-1 | Data on tumor inherence is still lacking, unfortunately. The population should be restricted to those having an ER+/HER2- tumor. By now the variable "targeted therapy" can be used as a surrogate for HER2-status.  Targeted therapy was delivered to 696 of 7116 patients of which 613 (88%) were found in the AI arm. HER2 status is a strong prognostic factor in ER+ disease and the skewed proportion of patients having targeted therapy, implicated as HER2 positive disease, inbetween the two strata of endocrine therapy can putatively explain the improved outcome by tamoxifen. This is real-world data - not a randomized trial - so all baseline data must evaluated.  The limitation of not being able to select patients by ER+/HER2- status should be elaborated on in the Discussion and I do recommend to exclude all patients having "targeted therapy" as a surrogate for HER2 status. | Thank you to the Reviewer for once again highlighting this critical suggestion. To address this significant issue, we have decided to repurchase the Health and Welfare Data Science Center data to obtain the ER and HER2 test results for breast cancer patients. In addition to the original study criteria, we added a restriction for ER+/HER2-, identifying 6,097 patients. Upon reviewing the medication use in this group, 47 patients were still found to have used targeted therapy. After excluding these, the number of study subjects is now 6,050.  This process required us to obtain a new IRB approval and undergo a review for the new data application, which has significantly extended the time required. Therefore, we would like to request an extension of the revision time once again. We appreciate your understanding and patience. |
| 6-２ | The Kaplan-Meier plots are to be complemented by a life-table showing numbers at risk. | With the suggestion of enhancing our Kaplan-Meier plots, we added a life-table displaying the number of at-risk patients. |
| 7 | PLOS authors have the option to publish the peer review history of their article ([what does this mean?](https://journals.plos.org/plosone/s/editorial-and-peer-review-process#loc-peer-review-history)). If published, this will include your full peer review and any attached files.  If you choose “no”, your identity will remain anonymous but your review may still be made public. | Yes. |
